# Monitoring the transition to open access through its mode of implementation: A principal component analysis of two surveys

**DOI:** 10.1371/journal.pone.0271215

**Published:** 2022-07-11

**Authors:** Keiko Kurata, Keiko Yokoi, Tomoko Morioka, Yukiko Minami, Masashi Kawai

**Affiliations:** 1 Department of Library and Information Science, Faculty of Letters, Keio University, Tokyo, Japan; 2 The Tokyo Foundation for Policy Research, Tokyo, Japan; 3 Kunitachi College of Music Library, Tokyo, Japan; 4 National Institute of Informatics, Tokyo, Japan; University of Siena, Italy, ITALY

## Abstract

Open access (OA) is transforming scholarly communication. Various modes of OA implementation have emerged, which reflect the complexity surrounding OA development. This study aimed to examine this development from the perspective of how OA is implemented. The sample comprised 2,368 randomly selected articles published in 2013 and 2,999 published in 2018 indexed in the Web of Science. We also conducted searches in Google and Google Scholar in 2015 for articles published in 2013 and in 2020 for articles published in 2018. Selected articles were categorized as either an “OA article,” “electronic subscription journal article,” or “not available online.” OA articles were classified into 10 implementation modes: Gold, Hybrid, Delayed, Bronze, Subject Repositories, Institutional Repositories, Personal/Institutional Websites, Academic Social Networks (ASNs), Others, and Web Aggregator. Overall, 56.5% of all sampled articles in 2013 were available for free on at least one website in 2015, while 61.7% of all sampled articles in 2018 were freely available on at least one website in 2020. Concerning implementation mode, ASNs had the highest frequency (44.4% in 2015 and 56.0% in 2020), followed by Subject Repositories (35.0% in 2015 and 39.6% in 2020) and Gold (24.1% in 2015 and 37.4% in 2020). To obtain an overview of OA implementation, we conducted principal component analysis with OA implementation mode as the variable for both 2015 and 2020. The first principal component was the axis indicating the number of overlapping OA implementations for each article in 2015 and 2020, while the second principal component was the axis orthogonal to the first, which was difficult to interpret. We identified three groups of OA implementation in each plot of the principal component scores for articles in 2015 and 2020; however, the OA implementation of each group differed in 2015 and 2020. This diversity reflects the respective positions of various stakeholders regarding OA.

## Introduction

Open access (OA) is transforming scholarly communication. In 2002, the Budapest Open Access Initiative defined OA as follows: “free availability [of journal articles] on the public internet, permitting any users to read, download, copy, distribute, print, search, or link to the full texts of these articles, crawl them for indexing, pass them as data to software, or use them for any other lawful purpose, without financial, legal, or technical barriers other than those inseparable from gaining access to the internet itself” (https://www.budapestopenaccessinitiative.org/read).

In BOAI, only Green road and Gold road were shown as the means to realize OA (OA implementation). The only OA implementation available at that time was the e-print archive (now arXiv) in the field of physics as Green road. There were only a few open access journals (OAJs) for the Gold road, and most of them were published by BioMed Central, a new publishing company that only publishes OA journals and is not a traditional academic publisher. The subsequent development of OA can also be considered as a situation in which the variety of available OA implementations expanded.

Next, we briefly discuss the characteristics of subject repositories and OA journals as typical OA implementations and then present some debates on Hybrid OA, which has emerged as a response to OA by traditional commercial publishers. In doing so, we provide an overview of how OA implementations emerge and are used in the context of diverse political, economic, and cultural factors related to scholarly communication and how this can lead to more complex situations at the same time.

PubMed Central (PMC), the leading repository of OA articles in the medical field, had 8 million articles archived as of May 2022. This OA implementation was popularized by the Public Access Policy, established by the NIH in 2004, which mandated that articles resulting from NIH-funded research be registered in PMC as OA. It is regarded as a successful representative example of the OA mandate policy by research funding agencies, which has dramatically influenced the promotion of OA.

The increasing number of OAJs, can be seen from the regularly published reports by Morrison on the Directory of Open Access Journals (DOAJ) [1, 2]. However, OAJs have diverse characteristics. Some are fully open access journals based on APCs from authors, competing with traditional commercial publishers such as the series of PLOS journals. Some others are OAJs that do not impose APCs (some researchers call them Diamond OA), and still others are OAJs such as *Nature Communication* published by traditional commercial publishers. DOAJ contains more than 14,000 journals, some of which have been awarded DOAJ Seals. According to the 2019 survey on DOAJ Seals (1390 journals), BioMed Central (Springer), Hindawi, MDPI, and SpringerOpen are the top four publishers in terms of journal titles, accounting for more than 60% of the total, while seven PLOS journals account for 19.6% of the papers published. The number of journals that did not impose APCs accounted for 28% of the DOAJ Seals [3].

Major commercial publishers have long considered subscription as the basis of scholarly publishing. One way of how they dealt with OA is the Hybrid OA that Springer launched in 2004 (https://www.springer.com/gp/about-springer/history). Hybrid journals are subscription journals that publish articles for which the author has paid the APC as OA. However, such a practice has been criticized as double-dipping in subscription fees and APCs [4], though Elsevier claims that both can be managed separately [5].

Hybrid OA has not been used much in OA implementation. However previous studies have used various methods. In studies with more than a certain sample size, the utilization rate of Hybrid OA is low. The highest rate was 8.3% by Piwowar et al. [6] in 2018, and the lowest was 0.5% by Borrego [7] in 2016. However, a survey report comparing the UK’s OA situation to the global situation shows that the percentage of Hybrid OA in articles written by UK authors in 2016 was 15.4%, nearly four times the global average of 4%. This situation has been caused by the UK’s OA policy, based on the Finch report published in 2012 [8], that mandates publication of publicly funded research results in OAJs. Furthermore, Hybrid OA has begun to impact current electronic journals’ contracts with academic libraries, even though it is still a small proportion of the total. Although BigDeal contracts between major commercial publishers and academic libraries are a mainstream practice, it is difficult for universities to support APC payments for many researchers as well as continue with BigDeal contracts for subscription journals. Therefore, the transformative agreements that combine subscription contracts and APC payments are becoming an essential topic of discussion among libraries and librarians [9].

Various other OA implementations have emerged, though it has been argued that some types of OA are not recognized as OA. For example, Harnad [10] argues that articles that are only accessible after an embargo period following publication should not be considered OA. Piwowar et al. [6] claimed that the Academic Social Networks (ASNs) such as ResearchGate should not be recognized as OA because they do not check for copyright compliance. Martín-Martín et al. [11] determined six dimensions that constitute OA: authoritativeness, user rights, stability, immediacy, peer review, and cost.

In this study, we aimed to understand OA’s current complicated situation by investigating the various methods, formats, and ways of implementing OA that are currently being used, including free access to full text, which was not been recognized as OA until recently. After conducting a survey of these various forms of articles available for free in 2015 and 2020, we compared the results to clarify how OA implementation has changed.

### Literature review

In our review of relevant literature, we selected studies that empirically investigated the mode of OA implementation, rather than those that explored the concept of OA. Table 1 shows the modes mentioned in previous studies (please refer to the S1 Table for detailed information, including discipline and sample size). The first column in Table 1 shows the 10 modes of OA implementation focused on in the current study (further details are described in the section “Categorization of modes of OA implementation”). “Closed” implies that the indicated study does not report on the mode of OA implementation. In the case of studies that conducted surveys using Unpaywall, the modes are consistent with Unpaywall’s settings; thus, these are categorized as Gold OA, Hybrid OA, Bronze OA, and Green OA. As such, only the oldest relevant study that used Unpaywall, that by Piwowar et al. [6], is shown in Table 1; the others are indicated in the table footnote.

**Table 1:** Comparison of modes of OA implementation.

Mode of OA implementation in this article	De Filippo & Mañana-Rodríguez (2020) [12]	Maddi (2020) [13]	Piwowar, Priem, & Orr (2019) [14]	Rovira, Urbano, & Abadal (2019) [15]	Martín-Martín et al. (2018) [16]	Piwowar et al. (2018) [6]	Universities UK (2017) [8]	Borrego (2016) [7]	Research Information Network (2015) [17]	Fathli, Lundén, & Sjögårde (2014) [18]	Archambault et al. (2013) [19]
Gold	DOAJ Gold	Gold	Gold	Open access journals	Gold OA	Gold OA	Gold—APC/Gold—no APC	Gold OA (APC journals/Free-of-charge journals)	Gold—APC/Gold—no APC	Gold	Gold OA
Hybrid	Other Gold	Closed	Hybrid	Hybrid journals or journals with embargo	Hybrid OA	Hybrid OA	Hybrid OA	Hybrid OA	Gold—Hybrid	Closed	Green and Hybrid OA
Delayed	Bronze	Bronze	Delayed Bronze		Delayed OA	Bronze OA	Delayed OA	Delayed OA	Delayed OA	Delayed	
Bronze			Immediate Bronze	Closed	Bronze OA		Closed	Complementary OA	Closed	Closed	
Subject Repositories	Green Accepted/Green Published	Green	Green	Subject-based repositories	Green OA	Green OA	Subject repository	Green OA	Subject repository	Closed	
Institutional Repositories				Repositories of linked institutions/Institutional repositories of other organizations/cooperative repositories			Institutional repository		Institutional repository	Green	
Personal/Institutional Websites	Closed	Closed	Closed	Personal websites belonging to researchers or research groups, projects websites/Other: websites owned by companies, associations, etc.	Freely Available	Closed	Academic	Gray OA	Academic	Closed	
Academic Social Networks				Academic social networks			ResearchGate/Social sharing network		Social sharing network		
Others				Orphan repositories			Filesharing/Other	Closed	Other		
Web Aggregators				Other: websites owned by companies, associations, etc.			Closed		Closed		Closed

Note: OA: open access; DOAJ: Directory of Open Access Journals; APC: Article Processing Charge

The following articles also used Unpaywall, so the mode of OA implementation is the same as those indicated for Piwowar et al. [6] in the table: Bosman & Kramer [20], European Commission [21], Van Leeuwen & Schneider [22], Morillo [23], Robinson-Garcia et al. [24], Singh et al. [25], Nishioka & Sato [26].

In early studies by Archambault et al. [19] and Fathli et al. [18], OA implementations were broadly classified, but most previous studies indicated five modes: Gold, Hybrid, Delayed, Bronze, and Green OA.

As for Personal/Institutional Websites and ASNs, there are disputes over whether these are considered OA. In Unpaywall, OA implementation is defined only for articles that can be mechanically identified as OA, such as having an OA license; articles that are ambiguous or cannot be uniformly identified are not considered OA, and consequently, they are considered “Closed” [6]. Surveys using the oaDOI data source behind Unpaywall treat Personal/Institutional Websites and ASNs similarly [12–14]. However, Martín-Martín et al. [16] consider any article that is freely accessible as “Freely Available” but do not consider these articles as Gold, Hybrid, Delayed, Bronze, or Green OA.

By contrast, some previous studies consider both Personal/Institutional Websites and ASNs as OA implementations based on their criteria and survey methods [7, 8, 15, 17]. Rovira et al. [15], for instance, created a very specific division of OA implementation. They subdivided Institutional Repositories into three modes: repositories of linked institutions, institutional repositories of other organizations, and cooperative repositories. Additionally, they included Web Aggregators, a relatively new service and rarely mentioned in previous studies, as an OA implementation. Although Rovira et al. [15] established detailed OA implementation modes, they limited their target to articles published by authors from CERCA research centers.

### Research questions

A variety of modes of OA implementation have been proposed and practiced thus far, reflecting the complex economic and political situation surrounding OA. Based on the comparison of previous studies that empirically investigated OA implementation, this study applies the following 10 modes of OA implementation, which are more detailed than those used in prior studies, and includes OA implementations that have not been universally recognized as OA. These OA implementations reflect the respective positions of various stakeholders, such as commercial publishers, academic societies, research funding agencies, and researchers, regarding OA.

This study aimed to better understand the current OA situation through its mode of OA implementation, which reveals the distinct stances or positions of the various stakeholders involved in OA.

We conducted two wide-ranging surveys that did not limit scope to the research output of specific institutions. The Web of Science database was selected to obtain bibliographic information of randomly selected articles published in 2013 and 2018, and Google and Google Scholar were also used to find full texts of target articles.

The study set the following research questions:

RQ1: What proportion of articles within the scope of this survey are freely available?

RQ2: Which mode of OA implementation is used most often for the surveyed articles?

RQ3: What changes occurred regarding the common modes of OA implementation between the 2015 and 2020 survey?

## Materials and methods

In this study, we (1) randomly selected the records of articles published in the same year from the Web of Science, (2) searched for the target articles using Google Scholar and Google, which are commonly used by researchers, and (3) visually inspected each web page and assigned the mode of OA implementation on the basis of the web page’s URL. (4) These websites were cross-checked between co-researchers. (5) To obtain an overview of the current state of OA implementation mode, we conducted principal component analysis with the OA implementations as the variable.

The Web of Science does not necessarily represent articles on a global scale but instead covers articles mainly published by traditionally highly regarded journals in English. It is somewhat biased toward journals subscribed to by commercial publishers rather than newly launched open access journals (OAJs). Therefore, the results of this survey may more strongly reflect the OA stance of major commercial publishers and academic societies in Europe and the United States. However, the current state of OA among major commercial publishers and academic societies is of great interest to many researchers, librarians, and policymakers, because it affects the future of scholarly communication.

Additionally, the search results using Google and Google Scholar may vary depending on when and where the article was searched for. Thus Google and Google Scholar have the limitations in terms of search reproducibility. However, numerous researchers now use Google Scholar, rather than specialized databases, to find and access articles [27]; therefore, we used both in this study.

### Sample of target articles

The survey aimed to ascertain the extent to which the full text of sampled articles published in 2013 (2,368 articles) and in 2018 (2,999 articles) was available for free as of January 2015 and January 2020, respectively. Records of samples were extracted randomly from the Web of Science. Since the most common embargo threshold is 12 months, we investigated the status of all sampled articles one year after publication.

The Web of Science consists of three databases, the Science Citation Index (SCI), the Social Sciences Citation Index (SSCI) and the Arts & Humanities Citation Index (A&HCI). For the survey in 2015, we purchased 2,000 records of randomly sampled articles from the SCI from Clarivate Analytics (which was Thomson Reuters at the time). From SSCI and the A&HCI, we extracted articles according to the ratio of the number of articles in the three databases, including the SCI (304 articles from SSCI, and 71 articles from A&HCI) by ourselves. Seven articles that were written by an anonymous author or that were simply commentaries on other articles were removed. The final sample comprised 2,368 articles.

For the survey in 2020, 3,000 records of randomly sampled articles in 2018 from the Web of Science were purchased from Clarivate Analytics. The document type was “Article,” and the databases constituted SCI-EXPANDED, SSCI, and A&HCI. As in the survey in 2015, one article with an anonymous author was excluded from the sample, so the final sample comprised 2,999 articles.

### Website search survey

First, we identified the URLs of journal websites on which articles were published using DOIs. We searched for articles without DOIs by journal title using Google.

Next, as the main survey, we searched for target articles using Google and Google Scholar, visually inspected each web page, and determined its status as “OA article,” “electronic subscription journal article,” or “not available online.” In the current study, the term “OA article” refers to full text being freely accessible. The search terms were a combination of the surname of the first author and the title of the article. We recorded the URLs of the first 10 results on Google and all results on Google Scholar and then checked whether the article’s full text was available. When the results of the Google or Google Scholar search did not include the URLs of the corresponding journal’s website identified through the preparation procedure, we additionally checked whether those websites were OA.

Articles whose full text were illegally provided for free by Sci-Hub—classified as pirate Black OA—did not appear in the search results in either survey. When a warning regarding security problem with found sites appeared on the PC screen, we did not continue attempting to access the website and recorded that there was no full text available on the website.

### Categorization of mode of OA implementation

Based on the literature review, we assigned 10 modes of OA implementation based on the URL where the full text of articles could be accessed. These 10 modes do not consider whether the link to the original data of the article require or not. We further classified the 10 modes into four groups according to the nature of the organization providing the articles for free.

The first group consists of Modes 1–4, in which articles are provided by academic journal publishers. The second group includes Modes 5 and 6, in which traditionally supported scholarly communication provides a system or location to make articles freely available. The third group included Modes 7, 8, and 9, which are considered problematic as the provider, or the provision method may not be consistent. ASNs (Mode 8) in particular were often not classified as OA implementation in previous studies. While assigning modes to the second and third groups, we did not check the permissions or licenses of the journals in which the articles were published. The last group, Mode 10, aggregates the full-text file of articles from various other websites and provides access to these articles in one place. This mode does not provide the link to the free full-text but to the files, but has not yet attracted much attention.

Group 1: Publishers

1. Gold: All articles in journals are openly available (free) on the publishers’ websites along with the latest issue (e.g., *PLOS ONE*). We only examined whether the full text was accessible from the journal publisher’s site, not whether the article processing charges (APCs) were paid.

2. Hybrid: Articles are immediately available for free through the subscription journal’s website based on the cost of the APC paid by the authors.

3. Delayed: Articles are available through the subscription journal’s website after an embargo period.

4. Bronze: Articles are temporarily available through the subscription journal’s website as an advertisement or are irregularly available for various reasons.

Group 2: Traditional organizations supporting scholarly communication

5. Subject Repositories: Articles are available from online archives containing research findings in a particular discipline (e.g., PMC, arXiv).

6. Institutional Repositories: Articles are available from online archives operated by universities and research institutions containing research findings by authors who belong to those institutions.

Group 3: Personal or other institutional provision of OA articles

7. Personal/Institutional Websites: Articles are available from websites operated by individual researchers or organizations other than universities and research institutions, such as non-profit organizations and companies.

8. ASNs: Articles are available from ASNs for researchers only (e.g., ResearchGate, Academia.edu).

9. Others: This category includes articles that are available for free on a website not intended to provide free access to articles but unintentionally makes these articles accessible: for example, websites such as the Internet Archive or EBSCO. EBSCO and ProQuest provide full text of articles for a fee; however, there are cases where an article’s full text can be accessed for free on their sites.

Group 4: New services aggregating OA articles provided by other websites

10. Web Aggregators: Websites aggregate OA article files from other websites and provide them in one place. These include Semantic Scholar, Dimensions, and Paperity.

### Mode of OA implementation and principal component analysis

For the principal component analysis, the correlation matrix—obtained from a matrix composed of articles as the object, OA implementation modes as the variable, and 1 (true) or 0 (false) as the observed value—was used as the data. Of the 10 principal components obtained, the first and second principal components, which have relatively large amounts of information, are presented in the next section as the analysis results.

## Results

### OA categories

Classifying the sampled articles into three categories resulted in the following: 56.5% (1,337) of all sampled articles published in 2013 were OA articles available on at least one website in 2015; 42.6% were electronic subscription journal articles available online for subscribers only; and 0.9% were not available online, indicating that the full text of an article was not available on the Internet. Of all sampled articles published in 2018, 61.7% (1,851) were OA articles available on at least one website in 2020. The percentage of both electronic subscription journal articles and not available online decreased to 38.1% and 0.2%, respectively (Table 2).

**Table 2:** Proportion of open access (OA) categories.

	2015		2020	
OA articles	1,337	56.5%	1,852	61.8%
Electronic subscription articles	1,009	42.6%	1,142	38.1%
Not available online	22	0.9%	5	0.2%
Total	2,368	100%	2,999	100%

### Modes of OA implementation

Table 3 shows the proportion of modes of OA implementation. The total percentage is over 100% because all modes included duplicates. For example, if an article was accessible from both PMC and *PLOS ONE*, we counted it for both Gold and Subject Repositories. However, if an article was accessible from both the first author’s and second author’s websites, it was counted only once as Personal/Institutional Websites. Therefore, the percentages in Table 3 are the percentages of each mode for all OA articles.

**Table 3:** Proportion of modes of open access (OA) implementation.

		2015 (n = 1,337)		2020 (n = 1,852)	
Publisher-hosted OA	1 Gold	322	24.1%	692	37.4%
	2 Hybrid	57	4.3%	153	8.3%
	3 Delayed	209	15.6%	172	9.3%
	4 Bronze	44	3.3%	88	4.8%
Green OA	5 Subject Repositories	468	35.0%	733	39.6%
	6 Institutional Repositories	187	14.0%	516	27.9%
Free availability	7 Personal/Institutional Websites	285	21.3%	213	11.5%
	8 Academic Social Networks	593	44.4%	1,038	56.0%
	9 Others	166	12.4%	297	16.0%
Access provided	10 Web Aggregators	10	0.7%	483	26.1%

ASNs had the highest frequency at 44.4% (593 articles) in 2015 and 56.0% (1,038 articles) in 2020. Their number doubled in 2020, and more than half of all OA articles were accessible from ASNs. ResearchGate accounted for 90.4% of OA articles from ASNs in 2015 and 96.1% in 2020. The second most common OA implementation mode was Subject Repositories, which accounted for 35.0% (468 articles) in 2015 and 39.6% (733 articles) in 2020. PMC was the most common repository, corresponding to 72.2% in 2015 and 72.9% in 2020. Next was arXiv, which accounted for 20.5% in 2015 and 21.1% in 2020. The others were CiteSeerx, EconStor, and SSRN.

Gold had the third-highest frequency at 24.1% (322 articles) in 2015 and 37.4% (692 articles) in 2020, a substantial increase. This order from first to third was the same in both surveys.

The top three OAJ titles were as follows: *PLOS ONE*, *Acta Physica Sinica*, and *Scientific Reports* in 2015, and *Scientific Reports*, *PLOS ONE*, *Nature Communications*, and *RSC Advances* in 2020 (*Nature Communications* and *RSC Advances* have the same number of articles). However, even *PLOS ONE* accounts for only about 10% of Gold, and Gold OA articles are distributed among many OAJs.

Gold includes OA articles accessible from OAJs distributed by publishers and platforms of non-profit organizations such as SciELO (Scientific Electronic Library Online), which supports academic journals making articles OA. SCOAP^3^ (Sponsoring Consortium for Open Access Publishing in Particle Physics) is also considered different from OAJ by publishers because the articles of supporting target journals are OA under the international collaboration of libraries and research institutions.

Institutional Repositories accounted for the most substantial increase in the percentage of total OA articles, from 14.0% (187 articles) to 27.9% (516 articles). Although most institutional repositories are operated by one specific organization (university or research institute), there are cases in which many universities and libraries form a consortium to jointly operate a single institutional repository, such as DiVA (Digitala Vetenskapliga Arkivet) in Sweden.

Bronze showed a slight increase in number but a difference in content. In 2015, many full texts of articles in the Bronze mode were samples provided by publishers for promotion. However, in 2020, new services such as Elsevier’s View Open Manuscript, Springer Nature’s SharedIt, and Cambridge University Press’s Cambridge Core Share, where the author’s version (i.e., preprint) is published on subscription journal sites, were found.

The number of OA articles of Web Aggregators grew from the small number of 0.7% (10 articles) in 2015 to 26.1% (483 articles). This rapid increase is due to the development of new forms of services such as Semantic Scholar, Dimensions, and Paperity.

An opposite trend was found for Delayed, which decreased from 15.6% (209 articles) to 9.3% (172 articles), and Personal/Institutional Websites, which also decreased substantially from 21.3% (285 articles) to 11.5% (213 articles).

### Number of modes of OA implementation used in articles

The number of modes of OA implementation for each sampled article is shown in Table 4. The use of all modes would equal 10; however, there were no such articles. Therefore, the maximum number of modes was six for four articles (0.3% of all OA articles) in 2015 and seven for two articles in 2020. For example, for ID32, a mathematics article published in *Publicacions Matemàtiques*, its OA implementation modes corresponded to Gold, Subject Repositories, Institutional Repository, Personal/Institutional Websites, ASNs, Others, and Web Aggregators. Furthermore, since this journal is not an OAJ, a subscription journal site was also found.

**Table 4:** Number of modes of open access (OA) implementation.

Number of modes of OA implementation	2015 (n = 1,337)		2020 (n = 1,852)	
1	719	53.8%	736	39.7%
2	349	26.1%	376	20.3%
3	180	13.5%	292	15.8%
4	65	4.9%	266	14.4%
5	20	1.5%	137	7.4%
6	4	0.3%	43	2.3%
7	0	0.0%	2	0.1%
Total	1,337		1,852	

The percentage of articles made OA through only one mode was 53.8% (719 articles) in 2015 and 39.8% (736 articles) in 2020. The percentage of articles accessible through multiple modes increased, and the average number of modes per article also increased from 1.75 (2015) to 2.37 (2020). Thus, we conclude that articles have been made more accessible.

### Articles made available through a single mode

#### Number of one-mode articles

The number of articles made available through a single OA implementation mode was calculated and is presented in Table 5. We recognized that the full text of articles provided for free only through publisher-hosted websites (Modes 1–4) indicates that only publishers as the original provider of the article offered OA articles. By contrast, articles made available only through websites other than publisher-hosted OA (Modes 5–10) indicate that the publisher’s site offers articles for a fee or that the publisher’s site is difficult to find on Google for some reason.

**Table 5:** Number of open access (OA) articles made available through a single mode.

		2015 (n = 719)		2020 (n = 736)	
Publisher-hosted OA	1 Gold	103	14.3%	83	11.3%
	2 Hybrid	14	1.9%	22	3.0%
	3 Delayed	77	10.7%	60	8.2%
	4 Bronze	24	3.3%	29	3.9%
Green OA	5 Subject Repositories	94	13.1%	89	12.1%
	6 Institutional Repositories	46	6.1%	117	15.9%
Free availability	7 Personal/Institutional Websites	97	13.5%	55	7.5%
	8 Academic Social Networks	237	33.0%	245	33.3%
	9 Others	27	3.8%	21	2.9%
Access provided	10 Web Aggregators	0	0%	15	2.0%
Total		719	100%	736	100%

In 2015, 237 articles (33.3% of all articles freely available through a single mode) corresponded to ASNs, followed by Gold (14.3%, 103 articles) and Personal/Institutional Websites (13.5%, 97 articles). In 2020, ASNs were the most popular mode, with 245 articles (33.3%) in 2015, followed by Institutional Repositories with 117 articles (15.9%) and Subject Repositories with 89 articles (12.1%).

#### Versions of articles available through a single mode

Among articles made OA through a single mode in 2020, the article versions available from the top three most frequent OA implementation modes—Academic Social Networks, Institutional Repositories, and Subject Repositories—were identified. We classified some article versions into two categories: publisher’s version and author’s version. The author’s version includes the accepted manuscript after peer review and the preprint before peer review. The manuscripts whose version could not be confirmed were classified as "not classified." When multiple versions of an article were found, the publisher’s version was given priority (Table 6).

**Table 6:** Versions available through a single mode in 2020.

	Subject Repositories		Institutional Repositories		Academic Social Networks	
Publisher’s version	9	10.1%	35	29.9%	175	71.4%
Accepted or author’s version	79	88.8%	79	67.5%	61	24.9%
Not classified	1	1.1%	3	2.6%	9	3.7%
Total	89	100%	117	100%	245	100%

PDFs of publisher’s versions were available for nine articles (10.1%) corresponding to Subject Repositories but for 175 articles (71.4%) corresponding to ASNs. On the other hand, the accepted or author’s version was available for 79 articles (88.8%) corresponding to Subject Repositories but for only 61 articles (24.9%) corresponding to ASNs. Institutional Repositories fell in the middle of the range.

Approximately 90% of the articles available only from Subject Repositories were the author’s version, which includes author-accepted manuscripts and preprints. However, that 70% of the articles are available only from ASNs are publisher’s versions suggests that authors may have mistakenly released the publisher’s version due to the complexity and diversity of copyright policies.

#### Characteristics of modes of OA implementation: Principal component analysis

**Fig 1:**
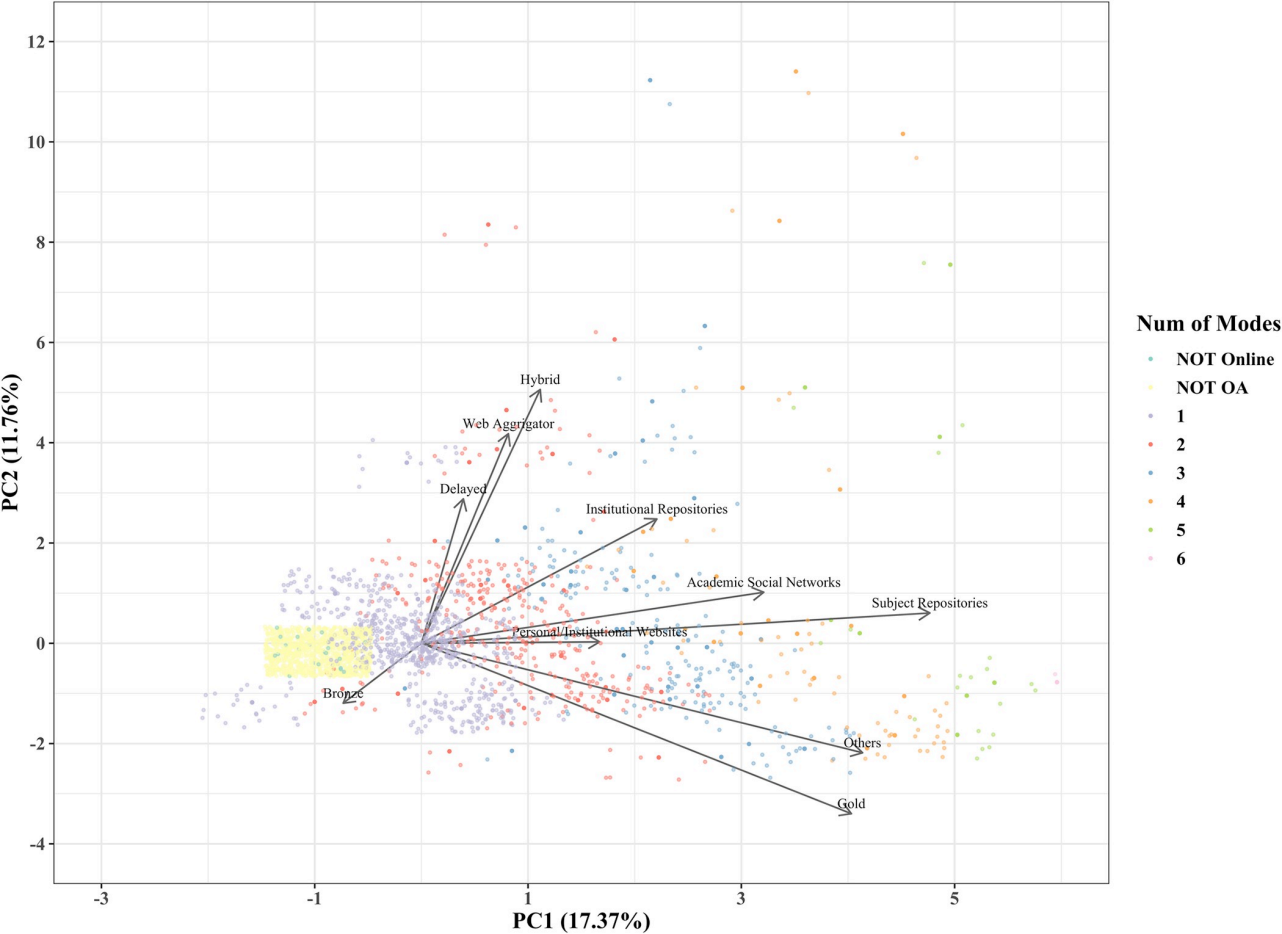
Principal component score plot (2015).

**Fig 2:**
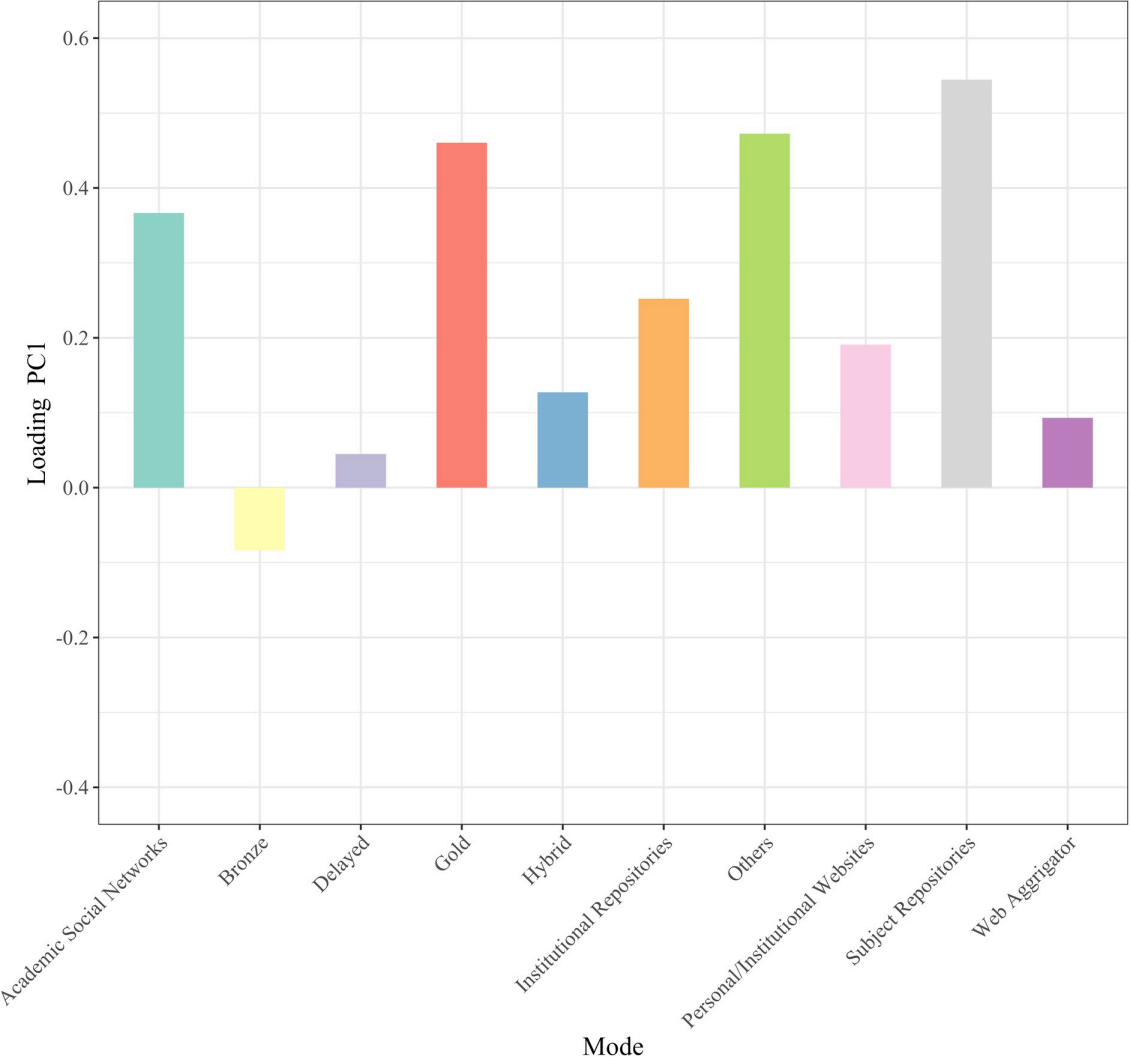
Principal component loadings (PC1 2015).

**Fig 3:**
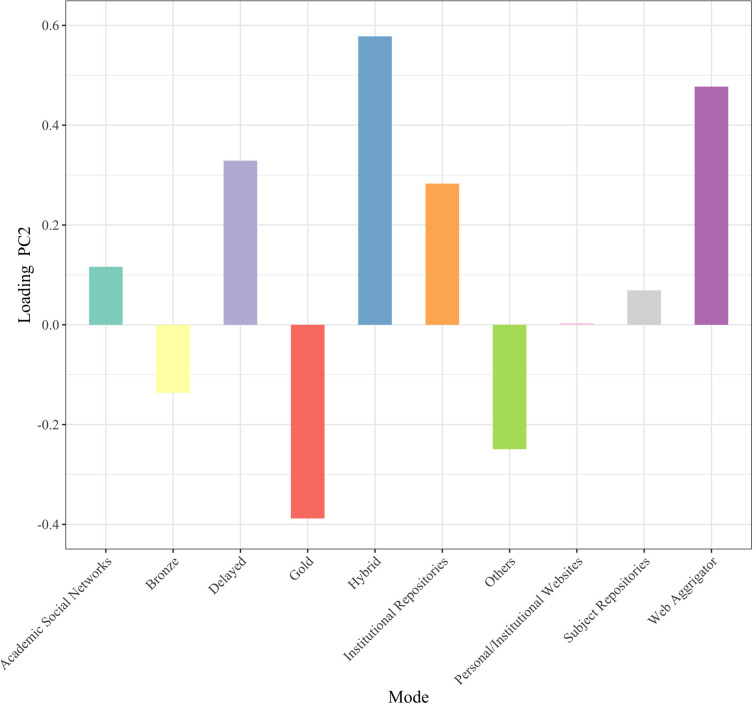
Principal component loadings (PC2 2015).

To obtain an overview of the current status of OA implementation modes, we conducted principal component analysis with mode of OA implementation as the variable. Fig 1 shows the plot of the principal component scores of articles in 2015. A principal component is a variable that is constructed as a mixture of the original variables and maximizes the variance among data points. The horizontal axis is the first principal component, the vertical axis is the second principal component, and the values in parentheses indicate the proportion of the variance. The green and yellow data points represent articles that are not available online and those not available for free, respectively. The number of modes used by each article is indicated by color: purple for one, red for two, blue for three, orange for four, yellow-green for five, and pink for six. The arrows indicate the principal component loadings of the first and second principal components. Since there were many data points with the same coordinate values, they were shifted slightly when plotted, and the transparency of the shifted data points was increased. Fig 2 shows the principal component loadings of the first principal components, and Fig 3 shows the same for the second principal components. The principal component loadings are the coefficients given to each of the original variables, which comprise the constitutive power for the principal components. The largest absolute principal component loadings of the first principal component are the value of Subject Repositories, followed by the value of Others and Gold, which mainly constitute the first principal component. The largest absolute principal component loadings of the second principal component were the value for Hybrid, followed by the values for Web Aggregators, Gold, and Delayed, which mainly constitute the second principal component.

In the first principal component, NOT OA is clustered around -1, articles made freely available through a single mode are concentrated around 0, and articles made freely available through more than three modes are distributed around the score exceeding 2. Based on this, the first principal component can be regarded as an axis that indicates the number of OA implementation modes used by an article; that is, how many duplicating modes are used.

Hybrid, Web Aggregators, Gold, and Delayed had large absolute principal component loadings in the second principal component, which was challenging to interpret on its own, especially for Web Aggregators, which included only 10 freely availably articles in 2015. However, we identified three groups located close to each other in Fig 1: 1) Hybrid, Delayed, and Web Aggregators; 2) Subject Repository and ASNs; and 3) Gold and Others. If we exclude Web Aggregators from the equation, the first group corresponds to OA articles provided in subscription journals. The second group includes articles that authors voluntarily make available for free in some way. The third group would include OAJ and services that provide freely available articles through a new mode, such as ScienceOpen.com, which is a platform that allows researchers to submit their research output and publishers to promote their journals.

**Fig 4:**
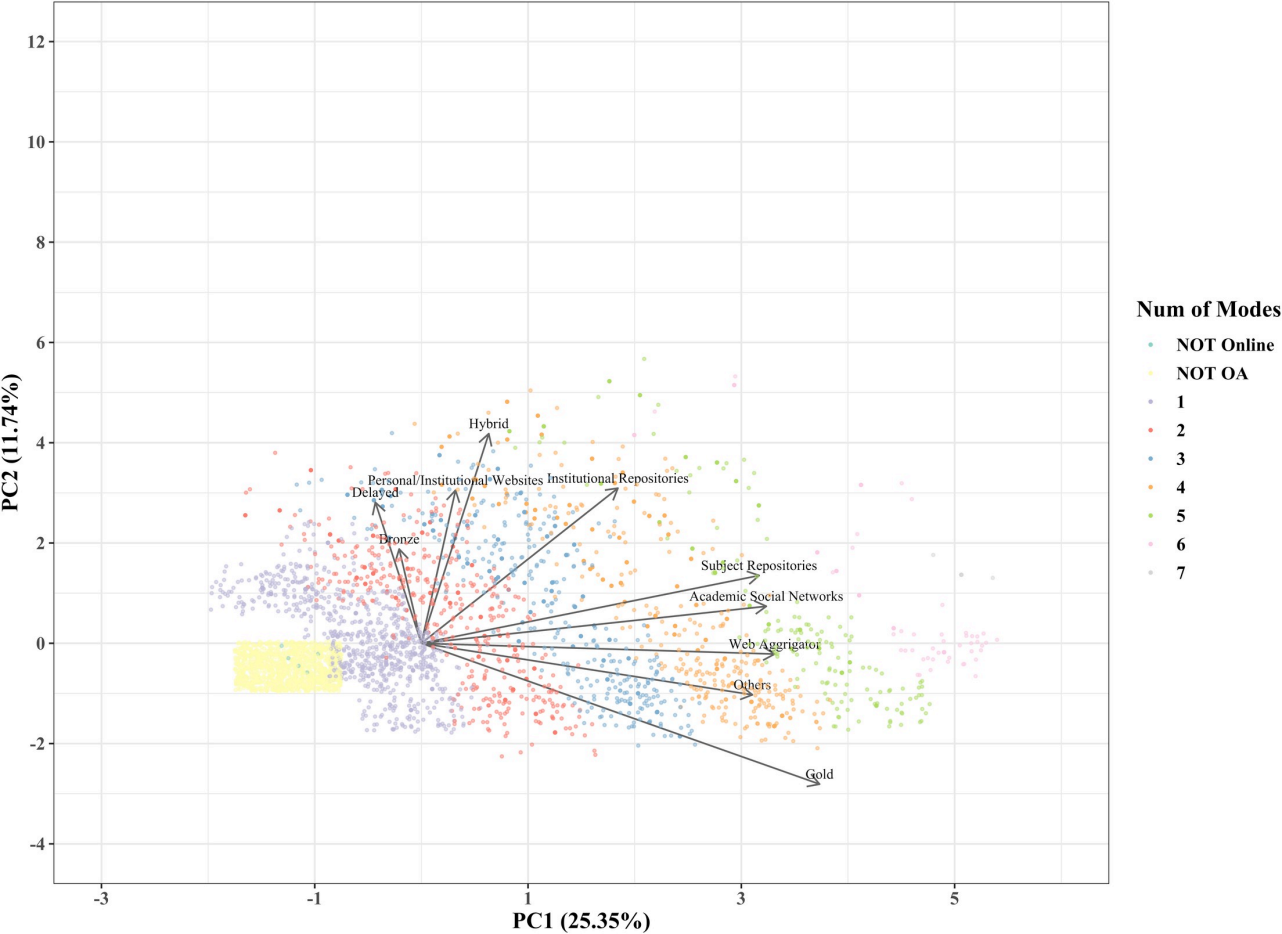
Principal component score plot (2020).

**Fig 5:**
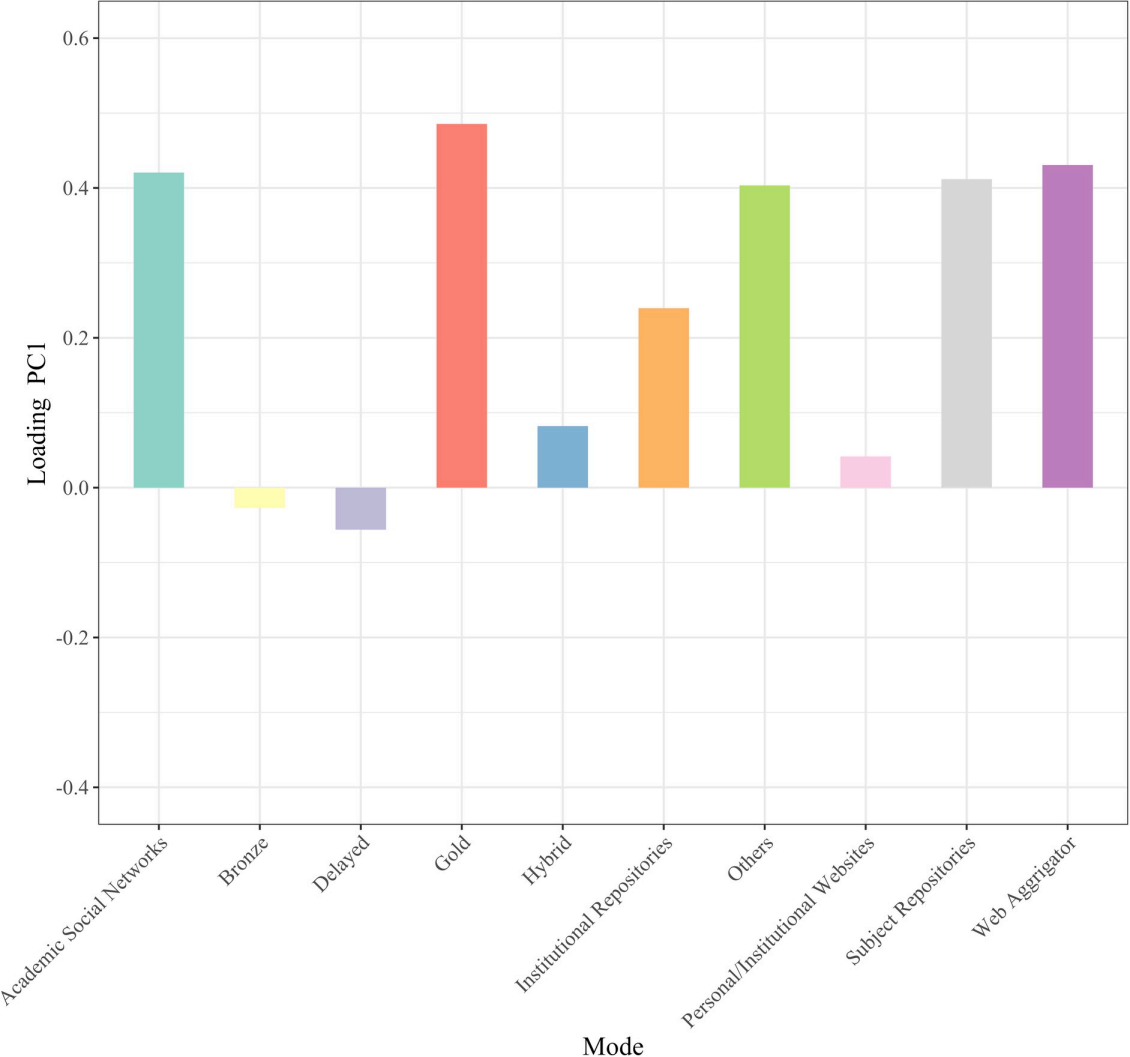
Principal component loadings (PC1 2020).

Fig 4 shows the same plot of the principal component scores of the articles in 2020. The colors used in Fig 4 indicate the same number of modes used for articles as in Fig 1, whereas gray was added to represent seven modes. In the plot, the first principal component is an axis indicating the degree of overlap of OA implementation modes as in 2015. The distribution of articles in Fig 4 is more extensive than that in Fig 1, indicating that the overlap of modes was stronger in 2020 than in 2015. As for the absolute principal component loadings of the first principal component, the value for Subject Repositories was the highest in 2015 but was replaced by Gold in 2020, followed by the values for Web Aggregators, ASNs, Subject Repository, and Others (Fig 5). This can be interpreted as the emergence of more overlapping articles centered around Gold.

**Fig 6:**
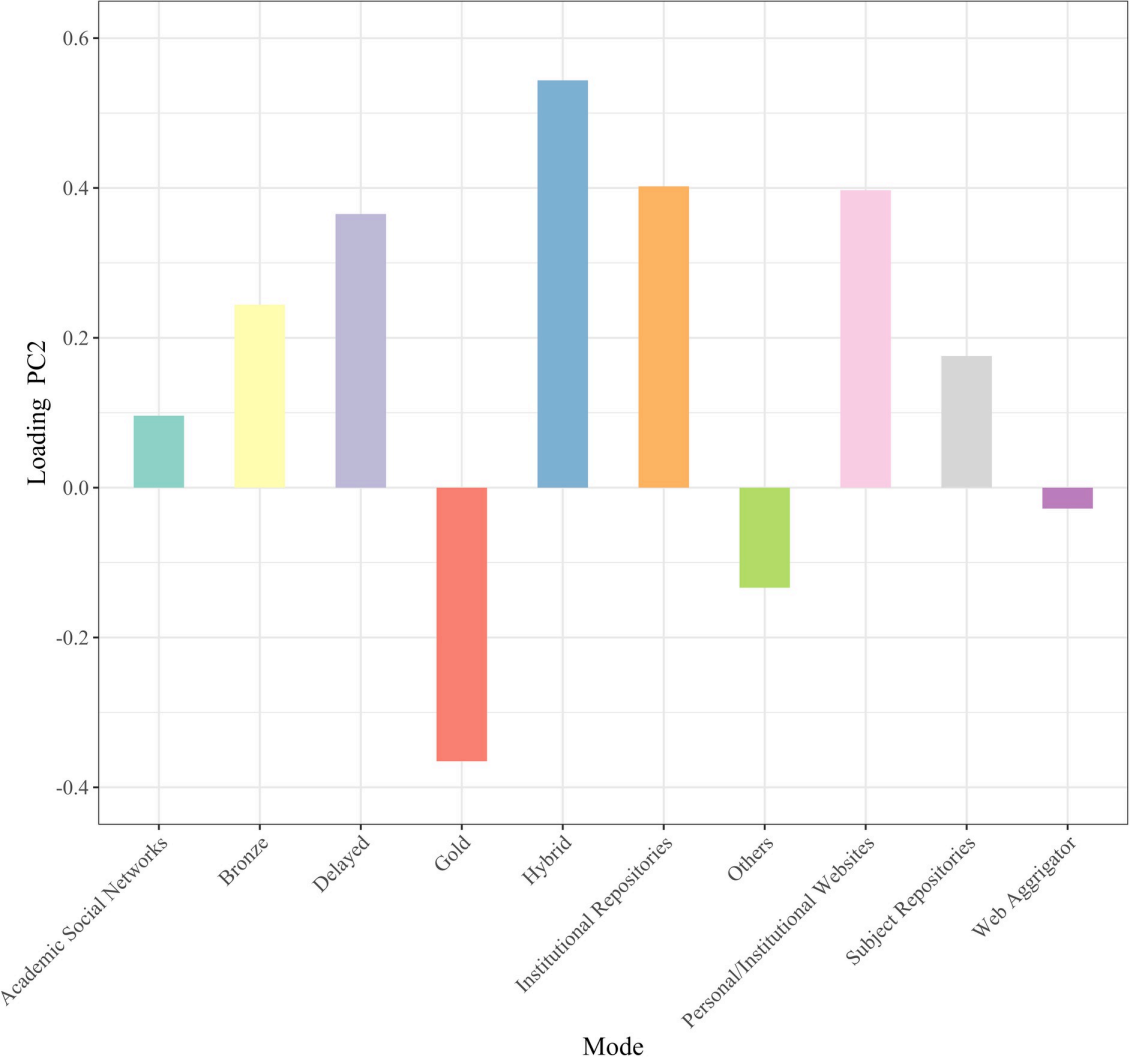
Principal component loadings (PC2 2020).

As for the second axis in 2020 (Fig 6), Gold stands out in the negative direction, which can be interpreted as OAJ emerging as a new form journal publishing. We can identify three groups located close to each other in Fig 4: 1) Hybrid, Delayed, Bronze, Personal/Institutional Websites, and Institutional Repositories; 2) Subject Repository, ASNs, Web Aggregators, and Others; and 3) Gold.

## Discussion

The overall percentage of freely available full text of articles was 56.5% in 2015 and 61.8% in 2020, higher than that reported by any previous study in all fields and populations. The current study found that ASNs were the most common mode: 44.4% of the articles were available from ASNs in 2015 and 56.0% in 2020. Additionally, 34.6% of all articles surveyed were also available on ASNs, followed by Subject Repositories (35.0% in 2015 and 39.6% in 2020) and Gold (24.1% in 2015 and 37.4% in 2020). The major features of the trend in OA implementation mode during 2015–2020 is the persistence of ASNs and the increase in the proportion of the Gold OA.

Principal component analysis was conducted to obtain an overview of OA implementation modes and their changes over time. The first principal component was interpreted as an axis showing the number of overlapping OA implementation modes in each article in 2015 and 2020. The second principal component as an axis was challenging to interpret for 2015 articles, but for 2020 articles, it constituted an axis that characterizes the new form of journal publishing as OAJ. Modes of OA implementation were classified into three groups for 2015 articles: 1) Gold and Others, 2) Hybrid and Delayed, and 3) Subject Repository and ASNs. For 2020, 1) Hybrid, Delayed, Bronze, Personal/Institutional Websites, and Institutional Repositories; 2) Subject Repository, ASNs, Web Aggregators, and Others; and 3) Gold were identified.

The changes from 2015 to 2020 are evident in four modes of OA implementation. First, Bronze did not correspond to any group in 2015 and was located in the same group as Hybrid and Delayed in 2020. In 2015, the articles corresponding to Bronze were published as samples in subscription journals for promotion by publishers, but in 2020, they were published as preprints of authors’ manuscripts as a new service. This is reflected in how Bronze changed in the principal component analysis.

Second, the position of Web Aggregator changed substantially. Although only 10 articles were available through web aggregators in 2015, the Web Aggregator mode was spread through multiple services (Semantic Scholar, Dimensions, and Paperity) in 2020. The establishment of Web Aggregator, which provides articles aggregated from other sites, as a mode is also considered the reason for the increase in OA implementation mode overlap in 2020.

Third, the position of Institutional Repositories was between 1) Subject Repositories and ASNs and 2) Hybrid and Delayed in 2015, but it corresponded to the position of the Hybrid, Delayed, Bronze, and Personal/Institutional Websites groups in 2020. Institutional Repositories is an OA implementation mode that expanded substantially from 2015 to 2020. However, we could not define any distinguishing characteristics, and thus we could not determine the meaning of this change in position.

Finally, Gold had the same negative direction on the vertical axis (second principal component) in the plots for 2015 and 2020. However, it was more negative in 2020, and thus we determined it should be considered a separate group from other modes. Gold showed the most substantial increase in the proportion of OA articles between 2015 and 2020, which suggests Gold OA may be positioned as a unique mode in the future.

At the beginning of the OA movement, only a few modes existed: Gold and Subject Repository. The present-day diversity of modes can be considered a sign of OA’s progress. A substantial feature of the current OA implementation situation is that ASNs, a controversial mode, whether it is considered OA implementation or not, provided the most free access to articles in our survey. There are many criticisms of this service regarding its uncertainty of copyright compliance and service stability [16, 28]. However, Springer Nature has started a trial program to provide ResearchGate articles published in Nature-branded journals from 2019, and the program has been extended based on the positive feedback from users [29]. If the collaboration between publishers and ASNs continues, a new service could be created, which may reshape the traditional definition of copyright and change the basic structure of OA.

The 2020 survey showed the Web Aggregator mode accelerated the accessibility of free articles, but it is uncertain whether this trend is sustainable. In addition, the proportion of OA articles increased for the Gold mode between 2015 and 2020. Although there are multiple reasons for this rise, the impact of Plan S may be undeniable, as this policy by research funding agencies mandating OA is driving the shift to OAJs. Moreover, since 2020, major commercial publishers and academic societies are beginning to enter into conversion agreements with libraries. This trend may increase the percentage of Hybrid and Gold OA modes.

This study discusses the development of OA from the perspective of OA implementation modes. OA is a movement that aims to fundamentally transform the traditional media of scholarly communication. In other words, it is an attempt to establish a new system and environment for the distribution of research. Therefore, the progress of OA is affected by recent technologies, services, policies, and trends that have emerged, and the mode of OA implementation reflects the diversity of OA scenarios.

It is likely that some OA implementation forms will cease to be used and that new modes will emerge in the future. Small but detailed surveys such as ours should be regularly conducted to define the characteristics of OA progress and discern changes in its development.

## Supporting information

S1 Table Previous studies that investigated mode of open access implementation in detail. (DOCX)

S1 File Data set. (XLSX)
