# Supplementary material for: Monitoring the transition to open access through its mode of implementation: A principal component analysis of two surveys
Source: PLoS One. 2022 Jul 11;17(7):e0271215. doi: 10.1371/journal.pone.0271215 (PMC9273081; doi:10.1371/journal.pone.0271215)
Supplement: S1 Table — (DOCX) [file pone.0271215.s001.docx]

**S1 Table. Previous studies that investigated modes of OA implementation in detail.**

| Study | Sample of documents | | | | | Method | OA rate  Total |
| --- | --- | --- | --- | --- | --- | --- | --- |
|  | Discipline | Source | Range | Pub. Year | Size |  |  |
| Archambault et al.  (2013) [1] | All disciplines | Scopus | Unlimited | 2004-2011 | 320,000  (40,000 per years) | Automated web-crawling | 38% - 44% |
| Fathli, Lundén, & Sjögårde (2014) [2] | All disciplines | SwePub | Refereed research articles from Swedish universities | 2011 | 23,905 | Checking against existing databases (DOAJ, Journal list of delayed OA, and information on IR registered in SwePub) | 25.4% |
| Research Information Network (2015) [3] | All disciplines | Scopus | Unlimited | 2010-2014 | Article, Review, Conference paper, Short survey | Combination of searching search sites and checking against existing databases | 27.3% |
|  | All disciplines | Scopus | Output of UK | 2010-2014 | Article, Review, Conference paper, Short survey | Combination of searching search sites and checking against existing databases | 35.0% |
| Laakso & Lindman (2016)[4] | Information Systems | Eight top journals | NA | 2010-2014 | 1515 | Manually searching Google and Google Scholar | 60% |
| Smith et al. (2017) [5] | Global Health | PubMed | Research articles indexed under the MESH “Global Health” | 2010-2014 | 3,366 | Combination of searching search sites and checking against existing databases | 58% |
| Teplitzky (2017) [6] | Earth science | Pangea | Articles whose data shared in data repository "Pangea" | 2010 | 744 | Combination of searching search sites and checking against existing databases | 75.4% |
|  |  |  |  | 2015 | 482 |  | 72.2% |
| Universities UK (2017) [7] | All disciplines | Scopus | Unlimited | 2016 | Article, Review, Conference paper, Short survey | Combination of searching search sites and checking against existing databases | 33% |
|  | All disciplines | Scopus | Output of UK | 2016 | Article, Review, Conference paper, Short survey | Combination of searching search sites and checking against existing databases | 53% |
| Martín-Martín et al. (2018) [8] | All disciplines | Web of Science | Unlimited | 2009 | 10,800,199 | Checking against existing databases | 53.1% |
|  |  |  |  | 2014 | 15,300,106 |  | 55.8% |
| Piwowar et al. (2018) [9] | All disciplines | Crossref | Unlimited | unlimited | 100,000 | Using oaDOI | 27.9% |
|  |  | Web of Science | Unlimited | 2009-2015 | 100,000 |  | 36.1% |
|  |  | Unpaywall | Unlimited | unlimited | 100,000 |  | 47.0% |
| Laakso & Polonioli (2018) [10] | Ethics | Institutional webpages, personal websites, PhilPapers profiles, and profiles on Google Scholar | Output of 297 researchers | 2010-2015 | 1,682 | Manually searching Google and Google Scholar | 3.4% - 23.5% |
| Bosman & Kramer (2018) [11] | All disciplines | Web of Science | Dutch 14 universities | 2010-2017 | 331,848 | Using Web of Science and oaDOI | 41.0% |
| Rovira, Urbano, & Abadal (2019) [12] | All disciplines | Web of Science | CERCA research centres | 2011-2015 | 3,730 | Manually searching Google and Google Scholar | 75% |
| European Commission (2019) [13] | All disciplines | Scopus | Unlimited | 2009-2018 | 15,491,402 | Using Unpaywall | 40.4% |
| Piwowar, Priem, & Orr (2019) [14] | All disciplines | Unpaywall | Unlimited | 1950-2019 | 71,000,000 | Using Unpaywall | 31% |
| Van Leeuwen & Schneider (2019) [15] | All disciplines | CWTS | R-Quest  5 universities in Denmark | 2014-2017/2018 | 854,548 | Using Web of Science and Unpaywall | 54% |
|  |  |  | R-Quest  5 universities in Norway |  |  |  | 53% |
|  |  |  | R-Quest  11 universities in Sweden |  |  |  | 53% |
|  |  |  | R-Quest  13 universities in the Netherland |  |  |  | 53% |
|  |  |  | R-Quest  48 universities in UK |  |  |  | 67% |
| Nishioka & Sato (2021) [16] | All disciplines | Unpaywall | Unlimited | unlimited | 79,534,697 | Using Unpaywall | 29.77% |
|  |  |  | Output of Japan | unlimited | 2,000,897 |  | 41.83% |
| Robinson-Garcia & van Leeuwen (2020) [17] | All disciplines | Web of Science | 963 universities identified in the Leiden Ranking database | 2014-2017 | 4,621,721 | Using Unpaywall | 40.7% |
| Singh, Piryani, & Srichandan (2020) [18] | All disciplines | Web of Science | Indian research output | 2014-2018 | 335,503 | Using Unpaywall | 24% |
| De Filippo & Mañana-Rodríguez (2020) [19] | All disciplines | Web of Science | Output of YERUN institutions | 2000-2019 | 453,573 | Using Web of Science | 32.0% |
| Morillo (2020) [20] | Economics | Web of Science | Articles written in English | 2017 | 20,030 | Using Web of Science | 14.5% |
|  | Immunology |  | Articles written in English | 2017 | 23,988 |  | 50.4% |
| Maddi (2020) [21] | All disciplines | OST | Unlimited | 2000-2017 | 27,500,000 | Using Web of Science | 31% |

1. Archambault E, Amyot D, Deschamps P, Nicol A, Rebout L, Roberge G. Proportion of Open Access peer-reviewed papers at the European and world levels: 2004–2011. Sciencemetrix. 2013. Available from: <http://www.science-metrix.com/pdf/SM_EC_OA_Availability_2004-2011.pdf>.

2. Fathli M, Lundén T, Sjögårde P. The share of Open Access in Sweden 2011 – analyzing the OA outcome from Swedish universities. Sciecom Info. 2014; 10. Available from <https://journals.lub.lu.se/sciecominfo/article/view/11645>.

3. Research Information Network. Monitoring the transition to open access: a report for the Universities UK Open Access Co-ordination Group; 2015. Available from: <https://www.universitiesuk.ac.uk/policy-and-analysis/reports/Documents/2015/monitoring-transition-to-open-access-2015.pdf>.

4. Laakso M, Lindman J. Journal copyright restrictions and actual open access availability: a study of articles published in eight top information systems journals (2010–2014). Scientometrics. 2016;109: 1167-1189. doi: [10.1007/s11192-016-2078-z](https://doi.org/10.1007/s11192-016-2078-z).

5. Smith E, Haustein S, Mongeon P, Shu F, Ridde V, Larivière V. Knowledge sharing in global health research—the impact, uptake, and cost of open access to scholarly literature. Health Res Policy Syst. 2017; 15:73. doi: [10.1186/s12961-017-0235-3](https://doi.org/10.1186/s12961-017-0235-3).

6. Teplitzky S. Open Data, [Open] Access: Linking Data Sharing and Article Sharing in the Earth Sciences. J Libr Sch Commun. 2017; 5:eP2150. doi:[10.7710/2162-3309.2150](https://doi.org/10.7710/2162-3309.2150).

7. Universities UK. Monitoring the transition to open access; 2017. Available from: <https://www.universitiesuk.ac.uk/policy-and-analysis/reports/Documents/2017/monitoring-transition-open-access-2017.pdf>.

8. Martín-Martín A, Costas R, van Leeuwen T, Delgado López-Cózar E. Evidence of open access of scientific publications in Google Scholar: a large-scale analysis. J Informetr. 2018;12: 819-841. doi:[10.1016/j.joi.2018.06.012](https://doi.org/10.1016/j.joi.2018.06.012).

9. Piwowar H, Priem J, Larivière V, Alperin JP, Matthias L, Norlander B, et al. The state of OA: a large-scale analysis of the prevalence and impact of Open Access articles. PeerJ. 2018;6: e4375. doi:[10.7717/peerj.4375](https://doi.org/10.7717/peerj.4375).

10. Laakso M, Polonioli A. Open access in ethics research: an analysis of open access availability and author self-archiving behavior in light of journal copyright restrictions. Scientometrics. 2018; 116: 291-317. doi:[10.1007/s11192-018-2751-5](https://doi.org/10.1007/s11192-018-2751-5).

11. Bosman J, Kramer B. Open access levels: a quantitative exploration using Web of Science and oaDOI data. PeerJ Prepr. 2018;6:e3520v1. doi: [10.7287/peerj.preprints.3520v1](https://doi.org/10.7287/peerj.preprints.3520v1).

12. Rovira A, Urbano C, Abadal E. Open access availability of Catalonia research output: case analysis of the CERCA institution, 2011-2015. PLoS ONE. 2019; 14: e0216597. doi:[10.1371/journal.pone.0216597](https://doi.org/10.1371/journal.pone.0216597).

13. European Commission. Trends for open access to publications. 2019 [Cited 2021 March 06]. Available from: <https://ec.europa.eu/info/research-and-innovation/strategy/goals-research-and-innovation-policy/open-science/open-science-monitor/trends-open-access-publications_en>

14. Piwowar H, Priem J, Orr R. The Future of OA: a large-scale analysis projecting Open Access publication and readership. bioRxiv. 2019. doi:[10.1101/795310](https://doi.org/10.1101/795310).

15. Van Leeuwen T, Schneider J . Open Access uptake of universities in the R-Quest countries under various OA mandates. In: Catalano G, Daraio C, Gregori M, Moed HF, Ruocco G, editors. Proceedings. 17th International Conference on Scientometrics and Informetrics, ISSI 2019. 2. Italy: “Sapienza” University of Rome; 2019. pp. 1560-1569. Available from: <https://www.issi-society.org/proceedings/issi_2019/ISSI%202019%20-%20Proceedings%20VOLUME%20II.pdf>.

16. Nishioka C, Sato S. The state of Open Access in Japan: An analysis using Unpaywall. Joho-Chishiki-Gakkaishi. 2021;31: 31-50. doi:[10.2964/jsik_2021_016](https://doi.org/10.2964/jsik_2021_016).

17. Robinson-Garcia N, Costas R, van Leeuwen TN. Open Access uptake by universities worldwide. PeerJ. 2020;8: e9410. doi:[10.7717/peerj.9410](https://doi.org/10.7717/peerj.9410).

18. Singh VK, Piryani R, Srichandan SS. The case of significant variations in gold–green and black open access: evidence from Indian research output. Scientometrics. 2020; 124: 515-531. doi:[10.1007/s11192-020-03472-y](https://doi.org/10.1007/s11192-020-03472-y).

19. De Filippo D, Mañana-Rodríguez J. Open access initiatives in European universities: analysis of their implementation and the visibility of publications in the YERUN network. Scientometrics. 2020;125:2667-2694. doi:[10.1007/s11192-020-03705-0](https://doi.org/10.1007/s11192-020-03705-0).

20. Morillo F. Is open access publication useful for all research fields? Presence of funding, collaboration and impact. Scientometrics. 2020;125: 689-716. doi:[10.1007/s11192-020-03652-w](https://doi.org/10.1007/s11192-020-03652-w).

21. Maddi A. Measuring open access publications: a novel normalized open access indicator. Scientometrics. 2020;124:379-398. doi:[10.1007/s11192-020-03470-0](https://doi.org/10.1007/s11192-020-03470-0).
